# Correlation of Clinical and Laboratory Features of Multisystem Inflammatory Syndrome in Children with Echocardiographic Findings

**DOI:** 10.3390/medicina61122158

**Published:** 2025-12-04

**Authors:** Nenad Barišić, Gordana Vijatov-Đurić, Borko Milanović, Ivana Vorgučin, Katarina Koprivšek, Milica Milojković, Ognjen Ležakov, Vesna Stojanović

**Affiliations:** 1 Faculty of Medicine, University of Novi Sad, Hajduk Veljkova 3, 21000 Novi Sad, Serbia; gordana.vijatov-djuric@mf.uns.ac.rs (G.V.-Đ.); borko.milanovic@mf.uns.ac.rs (B.M.); katarina.koprivsek@mf.uns.ac.rs (K.K.); milica.milojkovic@mf.uns.ac.rs (M.M.); ognjen.lezakov@mf.uns.ac.rs (O.L.);; 2 Institute for Child and Youth Healthcare of Vojvodina, Hajduk Veljkova 10, 21000 Novi Sad, Serbia

**Keywords:** multisystem inflammatory syndrome in children (MIS-C), high sensitive troponin I, blood urea nitrogen, fever, echocardiography

## Abstract

*Background and Objectives*: The aim of this study was to identify clinical features and laboratory findings that correlate with and predict pathological echocardiographic findings in children diagnosed with Multisystem Inflammatory Syndrome. *Materials and Methods*: Retrospective study included all patients aged 0–18 diagnosed with Multisystem Inflammatory Syndrome and hospitalized at our clinic from July 2020 to December 2022. The clinical and laboratory data of 61 patients were studied and compared between two subgroups (normal/abnormal echocardiography). *Results*: Elevated values of high-sensitivity troponin I were observed in 65.57% patients with MIS-C. The mean high-sensitivity troponin I value in the whole sample was 400.89 ± 1989.31 pg/mL. In patients with pathological echocardiographic findings, the mean value was 1240.24 ± 3609 pg/mL, while in patients with normal echocardiographic findings, it was 52.87 ± 71.86 pg/mL. Even though mean value was higher in the group of patients with abnormal echocardiography, no statistically significant difference was observed between high-sensitivity troponin I values in patients with and without pathological echocardiographic findings. Troponin levels were not in good correlation with pathological echocardiographic findings (point-biserial correlation; rpb = 0.25, *p* = 0.054) and were not good predictors of pathological echocardiographic findings (logistic regression; Chi^2^ = 3.77, *p* = 0.052). Logistic regression showed significant positive correlation of blood urea nitrogen levels and the degree of fever with abnormal echocardiographic findings (Chi^2^ = 10.04, *p* 0.002/Chi^2^ = 6.10, *p* = 0.013, respectively). *Conclusions*: Only blood urea nitrogen levels and high fever showed statistically significant correlation and predictive value for abnormal echocardiographic findings in children with Multisystem Inflammatory Syndrome. High sensitive troponin I had no significant power to discriminate between normal and abnormal echocardiographic findings.

## 1. Introduction

The emergence of Multisystem Inflammatory Syndrome in Children (MIS-C) as a severe post-infectious complication of SARS-CoV-2 focused significant attention to its diverse clinical manifestations, particularly cardiovascular involvement [1]. Left ventricular systolic dysfunction, mitral regurgitation, and coronary artery changes are prevalent echocardiographic findings in the acute phase of MIS-C [2]. Cardiac complications are frequently observed and contribute substantially to the morbidity and mortality of patients with MIS-C [2]. Those facts necessitate a thorough understanding of the clinical and laboratory parameters that correlate with and predict abnormal echocardiographic findings, enabling earlier identification and more targeted therapeutic interventions [2,3]. These cardiac manifestations can persist into the subacute phase [4]. Consequently, identifying reliable predictors for these echocardiographic abnormalities is crucial for effective risk stratification and long-term cardiac monitoring in affected children [4].

MIS-C is a hyper-inflammatory syndrome affecting individuals under 21 years of age, characterized by fever, laboratory evidence of inflammation, multi-organ involvement, and a recent SARS-CoV-2 infection or exposure [4]. While the exact pathogenesis remains under investigation, it is believed to involve a dysregulated immune response following SARS-CoV-2 infection [5]. This immune dysregulation often manifests as significant cardiac involvement, with echocardiographic abnormalities being a hallmark of the syndrome [4,6]. These cardiac manifestations can include ventricular dysfunction, coronary artery dilation, pericardial effusion, and valvular abnormalities which collectively underscore the critical need for detailed echocardiographic assessment in all suspected cases [4,5]. Early studies highlight that left ventricular ejection fraction often normalizes within weeks of acute illness; however, subclinical myocardial dysfunction, detectable by strain echocardiography, may persist for several months, necessitating ongoing vigilance [4]. Therefore, understanding the clinical and laboratory markers that predict these persistent cardiac anomalies is crucial for guiding appropriate follow-up care and mitigating long-term sequelae [4,7]. Given the variability in cardiac outcomes, ranging from complete resolution to persistent dysfunction, establishing robust predictive models is essential for stratifying risk and personalizing therapeutic strategies. For instance, elevated C-reactive protein levels and specific cardiac biomarkers like troponin have been consistently implicated as indicators of myocardial injury in acute MIS-C, necessitating further investigation into their long-term predictive utility for echocardiographic changes [2,5]. However, the precise correlation between the acute phase elevations of these markers and specific echocardiographic abnormalities at various follow-up stages remains an area requiring further elucidation [5]. Such investigations are vital for developing comprehensive guidelines for early detection and ongoing cardiac surveillance in pediatric patients with MIS-C [2,8]. Moreover, the transient nature of many cardiac complications in MIS-C, with resolution often observed within weeks to months, underscores the importance of longitudinal studies to differentiate temporary cardiac insults from those with potential long-term implications [9].

The aim of this study is to identify clinical features and laboratory findings that correlate with and predict pathological echocardiographic findings in children diagnosed with MIS-C, thereby contributing to improved diagnostic and prognostic strategies.

## 2. Materials and Methods

This retrospective study encompassed all patients aged 0–18 diagnosed with MIS-C and treated at our Institute from July 2020 to December 2022 and monitored over a subsequent one-year period after discharge from the hospital. Our Institute is the only tertiary-level pediatric institution with a pediatric intensive care unit (PICU) in the whole territory of Vojvodina Province and the only institution where pediatric patients diagnosed with MIS-C were treated. MIS-C diagnosis was established according to the WHO criteria [10]. Parents or guardians of all participants have signed a written informed consent for hospital admission and procedures performed during hospitalization. The study was approved by the Institute Ethics Committee and was conducted in accordance with the principles of the Declaration of Helsinki. Inclusion criteria: All previously healthy children diagnosed with MIS-C, aged 0–18 years, with available complete medical documentation (with all available clinical and laboratory findings, echocardiography findings). Exclusion criteria were as follows: incomplete documentation, not willing to participate in the study, previous diagnosis of chronic or inborn disease, and patients who received known cardiotoxic or nephrotoxic medications. All echocardiograms were performed and reviewed by two cardiologists using standardized image acquisition, standardized quantitative and qualitative assessments, and integrating multiple imaging techniques.

The patients were distributed into two groups: patients with normal and patients with pathological echocardiographic findings.

The data were obtained from patients’ medical records. A link to SARS-CoV-2 was established according to the positive antigen test to SARS-CoV-2 using oro/nasopharyngeal swabs or positive test to SARS-CoV-2 by reverse transcription polymerase chain reaction (RT-PCR) using oro/nasopharyngeal swabs at admission; positive serology test IgM and/or IgG for SARS-CoV-2); confirmed recent SARS-CoV-2 infection in the patient or his/her family members. SARS-CoV-2 IgM and IgG were detected using chemiluminescence immunoassay (CLIA). For all patients, demographic data (age, sex) and clinical data (maximal body temperature and duration of febrile periods) were collected. The data from the laboratory tests and findings were recorded, taking into account the age-specific reference ranges (blood counts, inflammatory markers, high sensitive troponin I, and markers of renal and liver function). The results of echocardiographic examinations were collected for all patients.

The clinical and laboratory data were compared between patients with normal and abnormal echocardiographic findings. This study employed a combination of descriptive and inferential statistical methods to analyze the data. In descriptive statistics, mean, median, and standard deviation were used for numerical variables, while frequency and/or percentage were applied for categorical variables. For testing normality of data distribution, we used Kolmogorov–Smirnov test. For the inferential statistics, we used standard statistical methods (Student’s *t* test, Mann–Whitney U test, Fisher’s exact test) as appropriate. For correlation and prediction testing, we used point-biserial correlation, Kendall’s Tau correlation, and logistic regression. For all calculations, *p*-value of 0.05 was considered statistically significant.

## 3. Results

The sample included 61 patients, of whom 24 (39.34%) were female and 37 (60.65%) were male.

The mean age of the participants was 7.24 years (SD ± 5.12 years). The age range of the participants was from 0.4 to 17.8 years.

Pathological echocardiographic findings were present in 29.51% (18/61) of the participants. Among the patients with pathological echocardiographic findings, there were 5 girls and 13 boys. No statistically significant difference in the frequency of abnormal echocardiographic findings between girls and boys was observed (Fisher’s exact test, *p* = 0.761).

Among patients with pathological echocardiographic findings, eight patients showed signs of pericarditis (pericardial effusion, pericardial edema with slight pericardial separation), two patients had newly detected isolated tricuspid valve regurgitation, four patients had newly developed isolated mitral valve regurgitation, and four patients showed signs of heart failure (of whom one patient also had pulmonary edema).

### 3.1. High-Sensitivity Troponin I

Elevated values of high-sensitivity troponin I (hs-TnI) above the reference limit were observed in 40/61 (65.57%) patients with MIS-C. The mean hs-TnI value in the whole sample was 400.89 ± 1989.31 pg/mL (min 1.5 pg/mL–max 14,260.9 pg/mL).

In patients with pathological echocardiographic findings, the mean hs-TnI value was 1240.24 ± 3609.20 (min 1.5 pg/mL–max 14,260.9 pg/mL, median 48.6 pg/mL), among whom 4/18 (22.22%) had normal values.

The mean hs-TnI value in patients with normal echocardiographic findings was 52.87 ± 71.86 pg/mL (median 36.00 pg/mL), with 17/43 (39.53%) having hs-TnI levels within the reference range.

Fisher’s exact test showed that there was no statistically significant difference in frequencies of normal hs-TnI levels between children with normal and abnormal echocardiography (*p* = 0.2461).

Even though the mean value was much higher in the group of patients with abnormal echocardiography, no statistically significant difference was observed between hs-TnI values in patients with and without pathological echocardiographic findings (Mann–Whitney U test, *p* = 0.287).

Kolmogorov–Smirnov test suggested that data for hs-TnI levels does not follow a normal distribution at the 0.05 significance level, so data was transformed using log transformation (Mean: 3.56, SD: ± 1.7, Minimum: 0.41, Maximum: 9.57, Quartile 1:2.52, Quartile 2:3.78, Quartile 3:4.34, Interquartile Range: 1.82, 95% Confidence interval for mean: 3.12–3.99). After log transformation, the data followed normal distribution (Kolmogorov–Smirnov test, D = 0.1465, *p* = 0.1319).

The results of the descriptive statistics showed that after log transformation, hs-TnI levels were still higher in the group of children with abnormal echocardiography (M = 4.20, SD = 2.23) than in the group with normal findings (M = 3.29, SD = 1.36). But even when normal distribution was achieved with log transformation, a two tailed *t*-test for independent samples showed that the difference between the groups was not statistically significant, (*p* = 0.054; 95% CI: −0.01–1.85).

A point-biserial correlation was run to determine the relationship between abnormal echocardiographic findings and hs-TnI values after log transformation. There was a positive correlation between abnormal echocardiographic findings and hs-TnI, but it was not statistically significant (rpb = 0.25, *n* = 61, *p* = 0.054).

A logistic regression analysis was performed to examine the influence of hs-TnI values after log transformation to predict the abnormal echocardiographic findings. Logistic regression analysis showed that the model as a whole was not significant (Chi^2^ = 3.77, *p* = 0.052, *n* = 61.00). The coefficient of the hs-TnI values after log transformation is b = 0.33, which is positive. This means that an increase in hs-TnI values is associated with an increase in the probability of abnormal echocardiographic findings. However, the *p*-value of 0.069 indicates that this influence is not statistically significant.

Hs-TnI levels were not dependent on patients’ age. The result of the Kendall’s Tau correlation showed that there was a negligible, positive correlation between age and Hs-TnI levels, but that correlation was not statistically significant, *p* = 0.357.

### 3.2. Clinical Features and Findings of Other Laboratory Tests

Clinical features and findings of other laboratory tests analyzed in our study are shown in Table 1. Kolmogorov–Smirnov test showed that all data sets presented in Table 1 were normally distributed.

Among all laboratory tests, only BUN levels were statistically different between children with normal and pathological echocardiographic findings (Table 1).

A two tailed t-test for independent samples showed that the difference in BUN levels between children with normal and abnormal echocardiographic findings was statistically significant, *t* (59) = 3.43, *p* = 0.001, 95% confidence interval [0.90–3.42]. The effect size (Cohen’s d) was 0.96, indicating that there was a large effect.

A point-biserial correlation was run to determine the relationship between abnormal echocardiography findings and BUN levels, and it showed statistically significant positive correlation (rpb = 0.41, *n* = 61, *p* = 0.001).

A logistic regression analysis was performed to examine the influence of BUN levels in prediction of abnormal findings. Logistic regression analysis showed that the model was significant (Chi^2^ = 10.04, *p* = 0.002, *n* = 61.00). The coefficient of the variable BUN was b = 0.41, which is positive. This means that an increase in BUN is associated with an increase in the probability of abnormal echocardiography. The *p*-value of 0.01 indicates that this influence is statistically significant. The odds Ratio of 1.51 indicates that one unit increase in the BUN levels will increase the odds that the dependent variable is 1 by 1.51 times.

**Figure 1:**
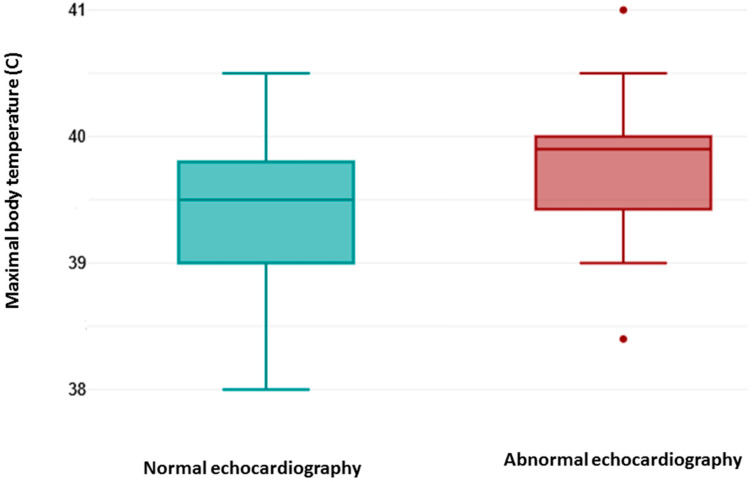
The maximal body temperatures measured during febrile episodes (fever).

As shown in Figure 1, the maximal body temperature measured during febrile episodes (fever) was higher in patients with abnormal echocardiography findings. A two tailed *t*-test for independent samples showed that difference between groups was statistically significant, t (59) = 2.43, *p* = 0.018, 95% confidence interval [0.07–0.76]. The effect size (Cohen’s d) was 0.68, indicating that there was a medium effect.

A point-biserial correlation was run to determine the relationship between abnormal echocardiography findings and fever. There was a positive correlation, which was statistically significant (rpb = 0.3, *n* = 61, *p* = 0.018).

A logistic regression analysis was performed to examine the influence of maximal body temperature in prediction of abnormal echocardiographic findings. Logistic regression analysis showed that the model as a whole was significant (Chi^2^ = 6.10, *p* = 0.013, *n* = 61). The coefficient of the independent variable is b = 1.24, which is positive. This means that an increase in maximal body temperature is associated with an increase in the probability of abnormal echocardiography. The *p*-value of 0.026 indicates that this influence is statistically significant. The odds ratio of 3.47 indicates that one unit increase in the maximal body temperature will increase the odds of abnormal echocardiographic finding by 3.47 times.

### 3.3. SARS-CoV-2 Serotypes

Distribution of pathological and normal echocardiographic findings among different serotypes of SARS-CoV-2 are shown in Table 2. 

In our sample, no statistically significant difference was found in the frequency of pathological echocardiographic findings among patients with different SARS-CoV-2 serotypes (Fisher’s exact test: Alpha vs. Delta *p* = 0.077, Alpha vs. Omicron *p* = 1.000, Delta vs. Omicron *p* = 0.063).

## 4. Discussion

This study investigated the relationship between laboratory markers, clinical features, and SARS-CoV-2 serotypes and echocardiographic findings in children with MIS-C. Pathological echocardiographic findings were present in 29.31% of the cohort. This prevalence aligns with other studies reporting significant cardiac involvement (20–60%) in MIS-C patients, emphasizing the need for comprehensive cardiac assessment in this population [3,11].

Although a higher proportion of boys had abnormal echocardiographic findings, this difference was not statistically significant, and this is consistent with studies showing only a slight male predominance without clear sex-related risk [12].

Elevated levels of hs-TnI were common (65.57% of patients). Despite higher mean of hs-TnI levels in the group with abnormal echocardiography, statistical analyses did not confirm a significant association between hs-TnI and echocardiographic abnormalities. Similar observations were made in several studies where troponin elevations did not reliably predict ventricular dysfunction or structural abnormalities [13,14,15].

This is in contrast with some prior studies that have reported associations between elevated troponin and cardiac dysfunction in MIS-C. However, other studies have also shown that troponin levels may not always correlate with the severity of cardiac manifestations in MIS-C, potentially reflecting the complex and multifactorial pathophysiology of cardiac involvement [2,16], The degree of myocardial inflammation, edema, and microvascular dysfunction may not be directly reflected in troponin release. The lack of association between hs-TnI and abnormal echocardiographic findings, despite frequent troponin elevation, could reflect the pathophysiology of MIS-C, where cardiac dysfunction may be driven by inflammation and cytokine storm rather than acute myocardial injury. This reinforces the concept that myocardial injury in MIS-C is multifactorial and that hs-TnI should not be used as a standalone indicator of clinically significant cardiac impairment.

In our study, BUN was the better predictor of abnormal echocardiographic findings. This highlights the potential role of renal biomarkers in risk stratification for cardiac complications in MIS-C, possibly indicating systemic inflammatory impact on multiple organ systems [4]. Elevation of BUN indicates that some degree of kidney involvement can have effect on cardiac manifestation of MIS-C. Several potential mechanisms could link renal involvement to cardiac dysfunction in MIS-C.

Elevated BUN and abnormal echocardiographic findings are both manifestations of more severe systemic illness in MIS-C and are often correlated through shared mechanisms (hypoperfusion, inflammation, and organ cross-talk). Elevated BUN can be a marker of higher risk for cardiac involvement and worse hemodynamics. There are serval possible mechanisms linking elevated BUN and cardiac dysfunction in MIS-C. Hypoperfusion/reduced cardiac output—myocardial dysfunction lowers cardiac output, causing renal hypoperfusion that triggers prerenal azotemia and elevated BUN. Conversely, volume depletion from fever/poor intake or capillary leak worsens both renal perfusion and preload, aggravating cardiac dysfunction. Thrombotic microangiopathy or microthrombi can impair renal microcirculation and coronary perfusion, linking renal dysfunction and myocardial injury. Low cardiac output activates sympathetic system and RAAS and thus promotes water and salt retention and induces proximal tubular reabsorption of BUN, while sympathetic activation drives distal tubular reabsorption of BUN. Heart dysfunction stimulates the elevation of antidiuretic hormone, which leads to the concentration of BUN by the medullary fraction of the kidney [17]. Also, one of mechanisms that may link elevated BUN and abnormal echocardiographic findings is cardiorenal syndrome type 5, where a systemic condition (in this case, MIS-C) causes concurrent dysfunction in both the heart and kidneys. The inflammatory milieu of MIS-C may directly affect both organs, leading to myocardial inflammation and reduced renal perfusion and function. Secondly, acute kidney injury itself can lead to fluid overload and electrolyte imbalances, which further strain the cardiovascular system and exacerbate cardiac dysfunction in already vulnerable patients [5]. Additionally, the systemic inflammatory response in MIS-C, characterized by cytokine storm, can directly injure both renal tubules and cardiac myocytes, thereby contributing to the observed associations between renal biomarkers and echocardiographic abnormalities [4]. Furthermore, evidence suggests that elevated cardiac biomarkers in MIS-C patients are associated with systolic dysfunction, but not necessarily with coronary artery abnormalities, implying a distinct pathophysiological mechanism involving diffuse myocardial stress rather than ischemic injury [2,18].

Secondly, acute kidney injury (AKI), even subclinical, can lead to volume overload and electrolyte imbalances, increasing cardiac workload and potentially exacerbating myocardial dysfunction. Moreover, the systemic inflammatory response characteristic of MIS-C can directly induce renal injury, thereby creating a vicious cycle that further compromises cardiovascular health [8].

All these findings suggest that a comprehensive assessment incorporating both cardiac and renal biomarkers is crucial for a distinct understanding of disease severity and for predicting echocardiographic abnormalities in pediatric MIS-C patients.

A statistically significant positive correlation was observed between pathological echocardiographic findings and the degree of fever, demonstrating some predictive value. Duration of fever, however, did not correlate with echocardiographic findings. This discrepancy suggests that the mere presence of fever, rather than its duration, might be a more immediate indicator of the acute inflammatory state that triggers cardiac involvement in MIS-C [4]. This aligns with the acute inflammatory nature of MIS-C, where rapid onset of systemic inflammation can swiftly impact cardiac function, independent of the protracted presence of fever. Such findings necessitate a deeper exploration into the temporal dynamics of inflammatory markers and their association with cardiac manifestations in MIS-C to better identify at-risk individuals early in the disease course. Further research could investigate the specific inflammatory mediators released during febrile episodes that might contribute to myocardial injury in MIS-C.

Pericarditis, valvular regurgitations, and signs of heart failure represented the primary cardiac abnormalities in our cohort. These findings closely match previously described patterns of reversible inflammatory myocardial involvement in MIS-C [12]. The transient nature of valve regurgitations and pericardial effusion reported in earlier studies further supports the hypothesis of inflammation-driven, reversible myocardial dysfunction rather than permanent myocardial injury.

No significant differences in the distribution of pathological echocardiographic findings were observed among children infected with Alpha, Delta, or Omicron variants. This aligns with recent reports showing that while acute COVID-19 severity varies by variant, MIS-C severity has remained relatively stable across multiple waves of the pandemic [19,20]. The present findings support the view that MIS-C is primarily driven by dysregulated immune responses rather than variant-specific viral characteristics.

Limitations of this study include the retrospective design, single-center setting, modest sample size—which reduces statistical power for subgroup comparisons, as well as the cross-sectional design which does not capture temporal evolution of cardiac or biochemical abnormalities. Due to all limitations, we acknowledge that our findings describe associations, not causation. Potential inter-observer variability in echocardiographic assessment should also be acknowledged.

## 5. Conclusions

Among the evaluated clinical and laboratory features of MIS-C, only BUN levels and high fever showed statistically significant correlation and predictive value for abnormal echocardiographic findings in children with MIS-C. Hs-TnI had no significant power to discriminate between normal and abnormal echocardiographic findings.

These findings underscore the importance of multimodal assessment in MIS-C and indicate that simple laboratory markers may aid in identifying children at risk for cardiac complications.

Future, larger multicenter studies are needed to confirm these associations and explore the potential of combining clinical risk scores with biomarker profiles to enhance the predictive accuracy for cardiac outcomes and provide a more comprehensive understanding of the complex interplay between clinical, laboratory, and echocardiographic findings in MIS-C.

## Figures and Tables

**Table 1:** Mean values ± SD of clinical features and laboratory tests in patients with normal and pathological echocardiographic findings.

Echocardiography:	Normal		Abnormal		
Variable	Mean Value or Frequencies	SD	Mean Value or Frequencies	SD	*p*
fever (max.temp) (C)	39.368	1.198	39.753	1.237	0.018
duration of fever (days)	6.86	3.76	6.94	3.35	0.08
ECG changes (yes/no)	3/38	/	4/16	/	0.202 *
SR (1/min)	68.25	27.19	55.13	25.38	0.131
CRP (mg/L)	188.49	96.26	151.86	73.52	0.154
WBC (×10^9^/L)	13.44	6.29	13.4	8.96	0.985
Hgb (g/L)	108.09	15.72	106.61	19.12	0.754
PLT (×10^9^/L)	308.42	141.54	291.89	128.75	0.671
Ferritin (μg/L)	2016.17	6091.7	973.00	1214.74	0.489
IL-6 (pg/mL)	190.11	253.61	678.92	1029.56	0.07
AST (μkat/L)	1.09	2.17	1.16	0.86	0.130
ALT (μkat/L)	0.95	1.65	0.95	0.73	0.986
GGT (μkat/L)	0.82	0.8	0.97	0.86	0.51
BUN (mmol/L)	3.66	2.05	5.82	2.67	<0.001
acidum uricum (μmo/L)	220.07	91.52	269.22	96.97	0.065
creatinine (μmol/L)	40.02	17.5	51.92	29.64	0.055
Fibrinogen (g/L)	6.15	1.7	5.93	1.23	0.614

Legend: ECG—electrocardiogram, SR—sedimentation rate, CRP—C-reactive protein, WBC—white blood cell count, Hgb—hemoglobin, PLT—platelets count, IL-6—interleukin 6, AST—aspartate aminotransferase, ALT—alanine aminotransferase, GGT—gamma-glutamyl transferase, BUN—blood urea nitrogen, *p*—*p*-value calculated with Student’s *t* test, *—*p*-value calculated with Fisher’s exact test.

**Table 2:** Distribution of pathological and normal echocardiographic findings among different serotypes of SARS-CoV-2.

	Pathological Echocardiographic Findings (No)		
SARS-CoV-2 Serotype	Yes	No	Total
Alpha	9	15	24
Delta	2	17	19
Omicron	7	11	18
total	18	43	61

## Data Availability

The data presented can be provided on request.

## Abbreviations

The following abbreviations are used in this manuscript:

MIS-C	Multisystem Inflammatory Syndrome in Children
hs-TnI	high sensitive troponine I
BUN	blod urea nitrogen
AKI	acute kidney injury

## References

[1] Sitthikool K., Junsawat P. (2024). Comparison of Clinical Features and Outcomes of Shocks in Multisystem Inflammatory Syndrome in Children Associated with COVID-19 (MIS-C), Septic Shock, and Cardiogenic Shock. Iran. J. Pediatr..

[2] Chakraborty A., Johnson J.N., Spagnoli J., Amin N., Mccoy M., Swaminathan N., Yohannan T., Philip R. (2022). Long-Term Cardiovascular Outcomes of Multisystem Inflammatory Syndrome in Children Associated with COVID-19 Using an Institution Based Algorithm. Pediatr. Cardiol..

[3] Brix N., Glerup M., Thiel S., Mistegård C.E., Skals R.G., Berntson L., Fasth A., Nielsen S., Nordal E., Rygg M. (2021). Proceedings of the 27th European Paediatric Rheumatology Congress (PReS 2021). Pediatr. Rheumatol..

[4] McAree D., Hauck A., Arzu J., Carr M., Acevedo J., Patel A.B., Husain N. (2022). Clinical Predictors of Subacute Myocardial Dysfunction in Multisystem Inflammatory Syndrome in Children (MIS-C) Associated with COVID-19. Pediatr. Cardiol..

[5] Diaz Y.B., Lattus A.F., Morales D.A.A., Díaz P.A. (2022). Compromiso cardiológico y marcadores inflamatorios en niños con Síndrome Inflamatorio Multisistémico relacionado a la infección por COVID-19. Andes Pediatr..

[6] Maaloul I., Gargouri R., Hadrich Z., Abid L., Kamoun T. (2023). Cardiac Involvement in Multisystem Inflammatory Syndrome in Children. Indian J. Pediatr..

[7] Truong D.T., Trachtenberg F.L., Hu C., Pearson G.D., Friedman K., Sabati A.A., Dionne A., Oster M.E., Anderson B.R., Block J. (2025). Six-Month Outcomes in the Long-Term Outcomes After the Multisystem Inflammatory Syndrome in Children Study. JAMA Pediatr..

[8] Abbas Q., Shahbaz F., Amjad F., Khalid F., Aslam M.N., Mohsin S. (2024). Short- and medium-term longitudinal outcomes of children diagnosed with multisystem inflammatory syndrome in children—Report from a single centre in Pakistan. Cardiol. Young.

[9] Kapoor R., Chandra T., Singh C.P., Singh R., Pandey I. (2022). Multisystem Inflammatory Syndrome in Children (MIS-C) Related to SARS-CoV-2 and 1-Year Follow-up. Indian J. Pediatr..

[10] World Health Organization (2020). Multisystem Inflammatory Syndrome in Children and Adolescents with COVID-19. https://www.who.int/publications/i/item/multisystem-inflammatory-syndrome-in-children-and-adolescents-with-covid-19.

[11] Kavurt A.V., Bağrul D., Gül A.E.K., Özdemiroğlu N., Ece I., Çetin I.I., Özcan S., Uyar E., Emeksiz S., Çelikel E. (2021). Echocardiographic Findings and Correlation with Laboratory Values in Multisystem Inflammatory Syndrome in Children (MIS-C) Associated with COVID-19. Pediatr. Cardiol..

[12] Belhadjer Z., Méot M., Bajolle F., Khraiche D., Legendre A., Abakka S., Auriau J., Grimaud M., Oualha M., Beghetti M. (2020). Acute Heart Failure in Multisystem Inflammatory Syndrome in Children in the Context of Global SARS-CoV-2 Pandemic. Circulation.

[13] Matsubara D., Kauffman H.L., Wang Y., Calderon-Anyosa R., Nadaraj S., Elias M.D., White T.J., Torowicz D.L., Yubbu P., Giglia T.M. (2020). Echocardiographic findings in MIS-C. J. Am. Coll. Cardiol..

[14] Jiang L., Tang K., Irfan O., Li X., Zhang E., Bhutta Z. (2022). Epidemiology, clinical features, and outcomes of multisystem inflammatory syndrome in children (MIS-C) and adolescents—A live systematic review and meta-analysis. Curr. Pediatr. Rep..

[15] Blatz A.M., Randolph A.G. (2022). Severe COVID-19 and multisystem inflammatory syndrome in children in children and adolescents. Crit. Care Clin..

[16] Zhao Y., Patel J., Huang Y., Yin L., Tang L. (2021). Cardiac markers of multisystem inflammatory syndrome in children (MIS-C) in COVID-19 patients: A meta-analysis. Am. J. Emerg. Med..

[17] Hillege H.L., Girbes A.R., De Kam P.J., Boomsma F., De Zeeuw D., Charlesworth A., Hampton J.R., Van Veldhuisen D.J. (2000). Renal function, neurohormonal activation, and survival in patients with chronic heart failure. Circulation.

[18] Kelly M.S., Fernandes N.D., Carr A.V., Beaute J.I., Lahoud-Rahme M., Cummings B.M., Chiu J.S. (2022). Diagnostic Yield of Cardiac Biomarker Testing in Predicting Cardiac Disease and Multisystem Inflammatory Syndrome in Children in the Pandemic Era. Pediatr. Emerg. Care.

[19] Levy N., Koppel J.H., Kaplan O., Yechiam H., Shahar-Nissan K., Cohen N.K., Shavit I. (2022). Severity and incidence of multisystem inflammatory syndrome in children during 3 SARS-CoV-2 pandemic waves in Israel. JAMA.

[20] Payne A.B., Gilani Z., Godfred-Cato S., Belay E.D., Feldstein L.R., Patel M.M., Randolph A.G., Newhams M., Thomas D., Magleby R. (2021). Incidence of Multisystem Inflammatory Syndrome in Children Among US Persons Infected with SARS-CoV-2. JAMA Netw Open..

